# Assessment of Selective and Universal Screening for Suicide Risk in a Pediatric Emergency Department

**DOI:** 10.1001/jamanetworkopen.2019.14070

**Published:** 2019-10-25

**Authors:** Jordan E. DeVylder, Taylor C. Ryan, Mary Cwik, Mary Ellen Wilson, Samantha Jay, Paul S. Nestadt, Mitchell Goldstein, Holly C. Wilcox

**Affiliations:** 1 Graduate School of Social Service, Fordham University, New York, New York; 2 Department of Mental Health, Johns Hopkins Bloomberg School of Public Health, Baltimore, Maryland; 3 Department of International Health, Johns Hopkins Bloomberg School of Public Health, Baltimore, Maryland; 4 Department of Psychiatry and Behavioral Sciences, Johns Hopkins School of Medicine, Baltimore, Maryland; 5 Division of Pediatric Emergency Medicine, Department of Pediatrics, Johns Hopkins School of Medicine, Baltimore, Maryland; 6 Department of Psychology, University of Maryland Baltimore County, Baltimore

## Abstract

**Importance:**

According to National Patient Safety Goal 15.01.01, all individuals being treated or evaluated for behavioral health conditions as their primary reason for care in hospitals and behavioral health care organizations accredited by The Joint Commission should be screened for suicide risk using a validated tool. Existing suicide risk screens have minimal or no high-quality evidence of association with future suicide-related outcomes.

**Objective:**

To test the association between results of the Ask Suicide-Screening Questions (ASQ) instrument in a pediatric emergency department (ED), implemented through selective and universal screening approaches, and subsequent suicide-related outcomes.

**Design, Setting, and Participants:**

In this retrospective cohort study at an urban pediatric ED in the United States, the ASQ was administered to youths aged 8 to 18 years with behavioral and psychiatric presenting problems from March 18, 2013, to December 31, 2016 (selective condition), and then to youths aged 10 to 18 years with medical presenting problems (in addition to those aged 8-18 years with behavioral and psychiatric presenting problems) from January 1, 2017, to December 31, 2018 (universal condition).

**Exposure:**

Positive ASQ screen at baseline ED visit.

**Main Outcomes and Measures:**

The main outcomes were subsequent ED visits with suicide-related presenting problems (ie, ideation or attempts) based on electronic health records and death by suicide identified through state medical examiner records. Association with suicide-related outcomes was calculated over the entire study period using survival analyses and at 3-month follow-up for both conditions using relative risk.

**Results:**

The complete sample was 15 003 youths (7044 [47.0%] male; 10 209 [68.0%] black; mean [SD] age, 14.5 [3.1] years at baseline). The follow-up for the selective condition was a mean (SD) of 1133.7 (433.3) days; for the universal condition, it was 366.2 (209.2) days. In the selective condition, there were 275 suicide-related ED visits and 3 deaths by suicide. In the universal condition, there were 118 suicide-related ED visits and no deaths during the follow-up period. Adjusting for demographic characteristics and baseline presenting problem, positive ASQ screens were associated with greater risk of suicide-related outcomes among both the universal sample (hazard ratio, 6.8 [95% CI, 4.2-11.1]) and the selective sample (hazard ratio, 4.8 [95% CI, 3.5-6.5]).

**Conclusions and Relevance:**

Positive results of both selective and universal screening for suicide risk in pediatric EDs appear to be associated with subsequent suicidal behavior. Screening may be a particularly effective way to detect suicide risk among those who did not present with ideation or attempt. Future studies should examine the impact of screening in combination with other policies and procedures aimed at reducing suicide risk.

## Key Points

**Question:**

Are results of universal and selective screening for suicide risk implemented in a pediatric emergency department associated with future suicidal behaviors?

**Findings:**

In this cohort study of 15 003 youths aged 8 to 18 years, positive screens were significantly associated with subsequent suicide-related hospital visits compared with standard emergency department procedures. Screening also more than doubled the detection of suicide risk compared with treatment as usual.

**Meaning:**

These findings suggest that screening for suicide risk in pediatric emergency departments is an effective approach to identify risk for subsequent suicide-related emergency department visits.

## Teaser

This cohort study examines the association between results of universal and selective screening for suicide risk in a pediatric emergency department using the Ask Suicide-Screening Questions (ASQ) instrument and subsequent suicide-related outcomes.

## Introduction

Suicide is the 10th leading cause of death in the United States and the 2nd leading cause of death among youth aged 10 to 19 years.^1^ Suicide incidence among adolescents has increased in the United States over recent decades, with pronounced increases among girls aged 10 to 14 years^2^ and black children aged 5 to 12 years.^3^ To successfully identify suicide risk, public health efforts must reach a broad proportion of those at risk for suicidal behavior.^4,5^ One approach is to screen for suicidal thoughts and behaviors in settings that may be accessed by individuals during high-risk periods,^6,7^ such as following emergency department (ED) discharge.^8,9^ The Joint Commission (TJC) released a Sentinel Event Alert^10^ recommending suicide risk screening in all individuals being treated or evaluated for behavioral health conditions as their primary reason for care in TJC-accredited hospitals and behavioral health care organizations and requires all health care organizations to use validated screening tools and other procedures for patients at risk of suicide as part of its accreditation process as of July 1, 2019. Despite the Sentinel Event Alert, many psychiatric EDs rely on clinical judgment of risk of harm to self and/or others^11,12^; medical EDs are even less objective and systematic.^13^ In addition, there are limited data on the predictive ability of the suicide risk screening tools recommended by TJC.

The Ask Suicide-Screening Questions (ASQ), a screening instrument that can be rapidly administered by hospital staff without specialized training in less than 2 minutes,^14^ could facilitate more widespread selective and universal screening approaches if shown to be associated with suicide-related outcomes compared with current procedures for identifying suicide risk. We previously found that the ASQ, implemented as a selective screening tool for youth presenting with behavioral or psychiatric presenting problems to a pediatric emergency setting, identified suicide risk among youth who had not otherwise reported suicidal thoughts or behaviors and that positive screens were associated with subsequent suicide-related return visits.^15^ Furthermore, the ASQ has been shown to identify high rates of undetected suicidal thoughts among youth aged 10 to 12 years.^16^ The ASQ has not yet been tested as a universal screen, although other universal screening methods have been found to be feasible with adult patients.^17^ While the ASQ has been screened in small convenience samples of patients with medical and psychiatric problems,^14^ to our knowledge, no study has reported on the association between results of universal suicide risk screening as routine care in a pediatric population and suicide-related outcomes.

The present study tested the association of positive ASQ screens with subsequent suicide-related outcomes among both a selective screening sample (ie, patients aged 8 to 18 years with a behavioral or psychiatric presenting problem) and a universal screening sample (ie, to patients aged 10 to 18 years with a medical presenting problem in addition to those aged 8 to 18 years presenting with a behavioral or psychiatric concern) in a pediatric ED. The aims were to (1) determine the association of ASQ screening results with subsequent return to the ED for suicide-related reasons or deaths by suicide; (2) evaluate whether the ASQ statistically improves on the association between the presenting problem and suicide-related outcomes; (3) determine whether the ASQ identifies risk among youth with no known risk of suicide; and (4) compare the ASQ’s effectiveness between universal and selective screening conditions.

## Methods

This was a retrospective cohort study of a consecutive case series of patients in the Johns Hopkins Hospital Pediatric ED from March 18, 2013, through December 31, 2018. Implementation was sequential, starting with selective screening of patients presenting to the ED for psychiatric or behavioral concerns in March 18, 2013, to December 31, 2016, and then broadened to universal screening in January 1, 2017, to December 31, 2018. Selective screening included patients aged 8 to 18 years with a behavioral or psychiatric presenting problem and the universal approach screened everyone aged 10 to 18 years in addition to those aged 8 to 18 years with behavioral or psychiatric problems. Nurses in the ED administered the ASQ as standard of care during triage (eAppendix 1 in the Supplement). The Johns Hopkins Pediatric ED is part of an urban academic pediatric medical center with approximately 30 000 patient visits per year. No patients were excluded on the basis of gender, minority status, or insurance type. If a patient screened positive on the ASQ, the ED physician was notified and patients were given additional evaluations and referrals as deemed appropriate, including emergency psychiatric evaluations when necessary, consistent with treatment as usual for potential suicide risk. The medical record review and evaluation of impact of suicide risk screening with the ASQ as routine care was approved by the Johns Hopkins School of Medicine institutional review board. Because screening was implemented as routine care, informed consent was not necessary. This study followed the Strengthening the Reporting of Observational Studies in Epidemiology (STROBE) reporting guideline for cohort studies. For further details on ED screening procedures, see eAppendix 2 in the Supplement.

### Screening Assessment

The ASQ is a 4-item nonproprietary suicide risk screening instrument that can be administered to patients in the pediatric ED by nurses, regardless of psychiatric training.^14^ A positive response to any of the questions is considered a positive screen, whereas a negative screen requires negative responses to all questions. In the initial development study, the ASQ was found to have a sensitivity of 96.9%, a specificity of 87.6%, and a negative predictive value of 99.7% for patients with medical and surgical concerns and 96.9% for patients with psychiatric concerns.^14^

### Measures

The primary exposure variable was binary, indicating a positive or negative ASQ screen at the index visit. Suicide-related presenting problem, based on the presence of suicidal ideation or attempt, was indicated with a binary variable. Presenting problems were mutually exclusive and limited to 1 problem per visit. Suicide-related presenting problems at the index visit were included in regression and survival models as an independent variable. Suicide-related presenting problems at follow-up visits were used as the outcome variable, combined with suicide deaths. To determine death by suicide, the ASQ database was matched with state death records of individuals aged 8 to 24 years between 2013 and 2018.

Additional measures abstracted from the electronic health record and included as covariates were demographic characteristics (age, gender, and race/ethnicity) and disposition at initial visit, which was collapsed into (1) discharged, (2) admitted or transferred, and (3) other, including leaving against medical advice. Race/ethnicity was coded as a single variable indicating non-Latino white, non-Latino black, Latino, or other.

### Statistical Analysis

#### Baseline Characteristics

To identify baseline characteristics associated with positive ASQ screens, respondents with positive and negative screens were compared on demographic variables (age, race/ethnicity, gender) and clinical variables (presenting problem, disposition) using χ^2^ analyses and *t* tests. These analyses were conducted separately for the universal and selective subsamples.

#### Survival Analyses

##### Universal and Selective Subsamples

Cox proportional hazards regression analyses were used to test for the risk of subsequent suicide death or ED visits for suicidal thoughts or behavior, defined based on electronic health record documentation of the presenting problem, using all available follow-up data. Follow-up period was determined based on number of days between the initial ED visit and either suicide death or subsequent ED visit due to suicidal ideation and/or behavior; in the absence of either of these events, data were censored as of December 31, 2018, the last date of available death record and clinical follow-up data. Survival analyses were conducted separately for the selective and universal subsamples, with adjustments in the first block of independent variables for sociodemographic characteristics (race/ethnicity and gender) and adjustments in the second block for baseline clinical variables (suicide-related presenting problem at index visit, disposition at index visit). Adjustment was made for the presenting problem to test whether the ASQ was associated with suicide-related outcomes above and beyond the presenting problem. An additional Cox regression of the entire sample with the inclusion of independent variables indicating universal vs selective screening, as well as an interaction term for universal screening × screening outcome, was run to test whether there was a significant difference in the association of positive ASQ screens with outcomes between the universal and selective screening conditions. All measures of association are presented as hazard ratios (HRs) with 95% confidence intervals and were considered significant at 2-tailed α = .05. Analysis was conducted using SPSS statistical software version 25 (IBM).

Unadjusted Kaplan-Meier survival curves are presented for data visualization, comparing time to subsequent suicidal behavior for positive vs negative screens. To illustrate the unique contribution of screening to the identification of risk for subsequent suicidal behavior, additional Kaplan-Meier survival curves were constructed to compare participants who (1) had negative screens and did not present with suicidal thoughts or behavior, (2) had negative screens but presented with suicidal thoughts or behavior, (3) had positive screens but did not present with suicidal thoughts or behavior, and (4) had positive screens and also initially presented with suicidal thoughts or behavior.

##### Psychiatric and Nonpsychiatric Universal Subsamples

An additional set of survival analyses were run to test the association of ASQ results with suicide-related outcomes among the universal screening subsample, divided into participants with and without a psychiatric presenting problem. The analyses among participants without psychiatric problems were intended to clarify whether the ASQ would be of value among participants presenting with a medical problem who would likely not be selectively screened for suicide risk.

#### Short-term Relative Risk

To be consistent with past literature,^7,8,9,18^ we focused on the risk period of 3 months following the initial visit. Among the 13 746 respondents with at least 3 months of follow-up, we calculated relative risk and risk differences associated with (1) positive ASQ screens, (2) suicidal thoughts or behavior as the initial presenting problem, and (3) the presence of either of the first 2 indicators (ie, to determine the relative risk based on an either-or scenario in which a respondent presented with either of the 2 indicators of suicide risk at baseline). Risk was calculated separately for the selective and universal groups and in the total composite sample, along with positive and negative predictive value, sensitivity, and specificity.

#### Exploratory Analyses 

Exploratory analyses included the following: (1) overall study procedures; (2) relative risk calculations for death by suicide; (3) demographic comparisons between selective and universal subsamples; (4) full frequency data for the short-term relative risk calculations; and (5) demographic comparisons between respondents who did vs did not present to the ED with suicidal behavior as their presenting problem, among those who then screened positive on the ASQ. In addition, relative risk was calculated using only death by suicide as an exploratory analysis, given the low prevalence of this outcome.

## Results

### Sample Characteristics

The ASQ was administered to a total of 15 003 participants (7044 [47.0%] male; 10 209 [68.0%] black; mean [SD] age, 14.5 [3.1] years at baseline) across the entire study period, with 4666 participants screened during the selective screening phase (2013-2016) and 10 337 screened during the universal screening phase (2017-2018). In the selective screening group, 2089 participants (44.8%) identified as male, and 3188 (68.3%) identified as black; mean (SD) age was 14.0 (3.1) years. Similarly, in the universal group, 4955 (47.9%) identified as male and 7021 (67.9%) as black; mean (SD) age was 14.7 (3.2) years. The selective and universal subsamples were temporally distinct and varied on several demographic factors (eFigure in the Supplement). Patients were followed up for a mean (SD) of 1133.7 (433.3) days in the selective screening condition and a mean (SD) of 366.2 (209.2) days in the universal condition. In the selective condition, there were 275 suicide-related ED visits and 3 deaths by suicide. In the universal condition, there were 118 suicide-related ED visits and no deaths during the follow-up period.

The protocol compliance rate (ie, percentage of eligible participants who were actually screened) ranged from 59% to 81% during the selective screening phase and 80% to 86% during the universal screening phase. Of all screened participants, 806 (7.8%) screened positive in the universal subsample, and 1435 (29.9%) screened positive in the selective subsample. Among both subsamples, positive screens were significantly more common among female respondents, respondents who were transferred or admitted, and those whose presenting problem was suicidal ideation or behavior (Table 1). The distribution of positive screens varied by race/ethnicity for the selective sample only, with white respondents most likely to screen positive and Asian respondents least likely (Table 1). There were no age differences in screening outcome for either sample (Table 1). Frequencies of positive screens and suicide-related presenting problems are shown in Table 2. In the combined sample, 150 out of 1012 youths (14.8%) who had a suicide-related presenting problem screened negative on the ASQ. Notably, 1229 of 2241 patients (54.8%) who screened positive on the ASQ did not report suicidal ideation or behavior as their presenting problem. This group, whose suicide risk may have otherwise been undetected, were disproportionately more likely to be male (61.0% of male patients with undetected risk vs 51.4% of female patients; χ^2^_1_ = 19.3; n = 2241; *P* < .001) or black (57.5% undetected) or Latino (54.5% undetected) compared with white (49.9%), other race (49.5%), or Asian (44.4%) (χ^2^_4_ = 11.7; n = 2241; *P* = .02). Undetected risk did not vary by age (detected: mean [SD], 14.3 [2.5] years; undetected: mean [SD], 14.2 [2.9] years; t_2239_ = 0.8; *P* = .45).

**Table 1.** Demographic and Clinical Descriptive Data by Positive ASQ vs Negative ASQ at Baseline Assessment

Characteristic	No. (%)		*P* Value
	Positive ASQ	Negative ASQ	
**Universal Sample**			
No.	806	9531	
Age, mean (SD)	14.7 (3.1)	14.8 (3.2)	.84
Race/ethnicity			
Black non-Latino	521 (7.5)	6444 (92.5)	.11
White non-Latino	197 (8.7)	2057 (91.3)	
Asian non-Latino	14 (9.9)	128 (90.1)	
Latino	55 (8.7)	580 (91.3)	
Other	19 (5.6)	322 (94.4)	
Gender			
Male^a^	310 (6.3)	4645 (93.7)	<.001
Female	496 (9.2)	4886 (90.8)	
Presenting problem			
Suicide related	361 (82.0)	79 (18.0)	<.001
Other	445 (4.5)	9452 (95.5)	
Disposition			
Discharged	516 (6.2)	7773 (93.8)	<.001
Admitted or transferred	280 (14.9)	1598 (85.1)	
Other	10 (5.9)	160 (94.1)	
**Selective Sample**			
No.	1435	3231	
Age, mean (SD)	13.98 (2.5)	14.06 (3.3)	.41
Race/ethnicity			
Black non-Latino	894 (28.2)	2274 (71.8)	<.001
White non-Latino	388 (38.6)	617 (61.4)	
Asian non-Latino	4 (22.2)	14 (77.8)	
Latino	57 (30.5)	130 (69.5)	
Other	92 (32.1)	195 (67.9)	
Gender			
Male	502 (24.0)	1587 (76.0)	<.001
Female	933 (36.2)	1644 (63.8)	
Presenting problem			
Suicide related	651 (90.2)	71 (9.8)	<.001
Other	784 (19.9)	3160 (80.1)	
Disposition			
Discharged	876 (24.7)	2671 (75.3)	<.001
Admitted or transferred	550 (52.3)	501 (47.7)	
Other	9 (13.2)	59 (86.8)	

Abbreviation: ASQ, Ask Suicide-Screening Questions.

^a^ Includes 3 transgender individuals (female to male).

**Table 2.** Cross-tabulation of ASQ Screening Results and Presenting Problems, by Screening Condition

Condition	No. (%)		Total No.
	Negative ASQ	Positive ASQ	
Selective			
Suicide-related problem	71 (9.8)	651 (90.2)	722
Other problem	3160 (80.1)	784 (19.9)	3944
Universal			
Suicide-related problem	79 (18.0)	361 (82.0)	440
Other problem	9452 (95.5)	445 (4.5)	9897

Abbreviation: ASQ, Ask Suicide-Screening Questions.

### Survival Analyses for Universal and Selective Subsamples

Both positive screens on the ASQ and suicide-related presenting problems at the index visit were significantly associated with subsequent visits for suicidal ideation or behavior (including death by suicide) in both the universal (HR, 6.8 [95% CI, 4.2-11.1]) and selective (HR, 4.8 [95% CI, 3.5-6.5]) screening conditions (Table 3). For the selective screening condition only, the likelihood of subsequent suicide-related ED visits varied significantly by race/ethnicity (HR for Latino vs non-Latino respondents, 1.7 [95% CI, 1.1-2.8]) and was higher for those who were admitted during their baseline visit (HR vs discharged, 1.4 [95% CI, 1.1-1.8]). Notably, the HR for a positive ASQ screen was numerically higher in the universal subsample compared with the selective subsample, and this subsample × screen interaction was statistically significant when tested in the full sample (Wald χ^2^_1_ = 6.2; *P* = .01). Kaplan-Meier curves illustrate the duration of follow-up until the first visit to the ED for suicide-related reasons, comparing both (1) respondents with positive vs negative screens on the ASQ and (2) all 4 combinations of presenting problem and screening outcome (Figure). When the universal subsample was divided based on whether the presenting problem was psychiatric vs nonpsychiatric, positive screens on the ASQ were associated with subsequent visits for suicidal ideation and behavior for both the psychiatric subsample (HR, 2.2 [95% CI, 1.2-4.1]) and the nonpsychiatric subsample (HR, 7.1 [95% CI, 3.2-15.8]) (eTable 1 in the Supplement).

**Table 3.** Cox Proportional Hazards Models of Association Between ASQ Screens and Subsequent Suicide-Related Outcomes

Variable	HR (95% CI)	
	Model 1^a^	Model 2^b^
**Universal Sample**		
ASQ result		
Positive	11.2 (7.8-16.1)	6.8 (4.2-11.1)
Negative	1 [Reference]	1 [Reference]
Race/ethnicity		
Black non-Latino	1 [Reference]	1 [Reference]
White non-Latino	0.9 (0.6-1.4)	0.9 (0.5-1.4)
Asian non-Latino^c^	NA	NA
Latino	1.4 (0.8-2.8)	1.5 (0.8-2.8)
Other	0.6 (0.1-2.4)	0.5 (0.1-2.1)
Gender		
Male^d^	0.9 (0.6-1.2)	0.8 (0.6-1.2)
Female	1 [Reference]	1 [Reference]
Presenting problem		
Suicide related	NA	2.2 (1.3-3.8)
Other	NA	1 [Reference]
Disposition		
Discharged	NA	1 [Reference]
Admitted or transferred	NA	1 [Reference]
Other	NA	0.7 (0.1-4.9)
**Selective Sample**		
ASQ result		
Positive	6.1 (4.7-8.0)	4.8 (3.5-6.5)
Negative	1 [Reference]	1 [Reference]
Race/ethnicity		
Black non-Latino	1 [Reference]	1 [Reference]
White non-Latino	0.9 (0.7-1.2)	0.9 (0.6-1.1)
Asian non-Latino	1.6 (0.2-11.2)	1.5 (0.2-10.6)
Latino	1.7 (1.1-2.8)	1.7 (1.1-2.8)
Other	0.8 (0.5-1.4)	0.8 (0.5-1.4)
Gender		
Male^d^	0.7 (0.6-0.9)	0.8 (0.6-1.0)
Female	1 [Reference]	1 [Reference]
Presenting problem		
Suicide related	NA	1.4 (1.1-1.9)
Other	NA	1 [Reference]
Disposition		
Discharged	NA	1 [Reference]
Admitted or transferred	NA	1.4 (1.1-1.8)
Other	NA	NA

Abbreviations: ASQ, Ask Suicide-Screening Questions; HR, hazard ratio; NA, not applicable.

^a^ Model was adjusted for demographic characteristics.

^b^ Model was adjusted for demographic characteristics, presenting problem, and disposition.

^c^ Hazard ratios could not be calculated for the Asian non-Latino subgroup because there were no suicide-related follow-up visits among the Asian non-Latino participants in the universal screening subsample.

^d^ Includes 3 transgender individuals (female to male).

**Figure.**
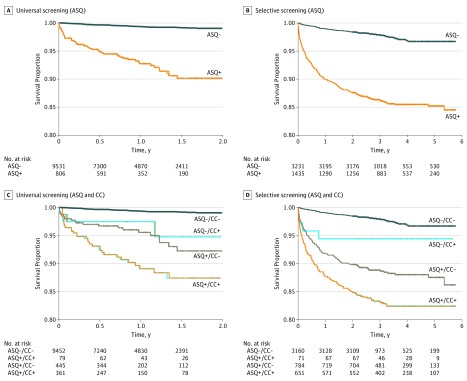
Kaplan-Meier Survival Curves Comparing Time Until Suicide Outcomes Following the Index Visit A and B, Respondents who screened positive on the Ask Suicide-Screening Questions (ASQ+) in the universal screening condition (A) and selective screening condition (B) had a shorter time to suicide-related outcome than those who screened negative (ASQ−). C and D, Respondents with ASQ+ and presenting problems related to suicide (CC+; ie, suicidal ideation or behavior) had the shortest time to suicide-related outcome in the universal screening (C) and selective screening (D) conditions compared with all other combinations. CC− indicates respondents whose presenting problem was not related to suicide.

### Short-term Relative Risk

Relative risk was calculated based on the likelihood of returning to the ED for suicide-related problems within 3 months of the initial index visit. For the selective subsample, universal subsample, and combined total sample, a positive ASQ screen combined with a suicide-related index problem yielded the greatest relative risk, followed by a positive ASQ screen alone, and finally by a suicide-related index presenting problem with a negative ASQ screen (Table 4). Positive and negative predictive values were similar between screening and presenting problem approaches to identifying suicide risk, although screening consistently yielded higher sensitivity but lower specificity (Table 4). For complete cross-tabulated data, see eTable 2, eTable 3, and eTable 4 in the Supplement.

**Table 4.** Relative Risk and Related Properties of Positive ASQ Screens, Based on a 3-Month Follow-up Period

Condition or Variable	Selective	Universal	Full Sample
ASQ			
RR (95% CI)	12.4 (7.0-21.8)	18.2 (10.1-32.9)	17.8 (12.0-26.3)
Sensitivity (95% CI)	84.6 (75.5-91.3)	60.0 (44.2-74.3)	76.5 (68.4-83.3)
Specificity (95% CI)	70.3 (69.0-71.6)	92.7 (92.1-93.2)	85.1 (84.5-85.7)
PPV (95% CI)	5.4 (4.9-5.9)	3.9 (3.1-5.0)	4.9 (4.4-5.4)
NPV (95% CI)	99.6 (99.3-99.7)	99.8 (99.7-99.9)	99.7 (99.6-99.8)
Positive likelihood ratio	2.9	8.2	5.2
Negative likelihood ratio	0.2	0.4	0.3
Presenting problem			
RR (95% CI)	5.8 (3.9-8.7)	17.1 (9.6-30.7)	10.8 (7.8-15.0)
Sensitivity (95% CI)	51.7 (40.9-62.3)	42.2 (27.7-57.9)	48.5 (39.9-57.3)
Specificity (95% CI)	85.3 (84.2-86.3)	96.0 (95.6-96.4)	92.4 (91.9-92.8)
PPV (95% CI)	6.5 (5.3-7.9)	5.0 (3.5-7.0)	6.0 (5.0-7.1)
NPV (95% CI)	98.9 (98.6-99.1)	99.7 (99.6-99.8)	99.5 (99.4-99.5)
Positive likelihood ratio	3.5	10.5	6.4
Negative likelihood ratio	0.6	0.6	0.6
ASQ or presenting problem			
RR (95% CI)	15.3 (8.2-28.6)	19.8 (10.8-36.2)	20.4 (13.3-31.1)
Sensitivity (95% CI)	87.9 (79.4-93.8)	64.4 (48.8-78.1)	80.2 (72.5-86.5)
Specificity (95% CI)	68.8 (67.5-70.2)	91.9 (91.3-92.4)	84.1 (83.5-84.7)
PPV (95% CI)	5.3 (4.9-5.8)	3.8 (3.1-4.7)	4.8 (4.4-5.2)
NPV (95% CI)	99.7 (99.4-99.8)	99.8 (99.7-99.9)	99.8 (99.7-99.8)
Positive likelihood ratio	2.8	7.9	5.1
Negative likelihood ratio	0.2	0.4	0.2

Abbreviations: ASQ, Ask Suicide-Screening Questions; NPV, negative predictive value; PPV, positive predictive value; RR, relative risk.

### Death by Suicide

There were 3 deaths in the selective screening subsample and none in the universal subsample. The relative risk for confirmed death by suicide in the selective screening subsample was 4.5 (95% CI, 0.4-49.6). For further detail, see eAppendix 3 in the Supplement.

## Discussion

### Main Findings

Our study found that positive screens on the ASQ in a pediatric ED were associated with subsequent suicidal behavior, defined as either suicide-related ED visits or death by suicide, and that this association was both statistically independent of, and of greater magnitude than, the association of the index presenting problem alone with suicide-related outcomes. The ASQ was particularly effective at identifying risk for subsequent suicide-related ED visits among patients who had no psychiatric presenting problems during their index visit. While there were only 3 confirmed deaths by suicide in this cohort, 2 of the participants who died had screened positive on the ASQ, translating to a statistically nonsignificant relative risk of 4.5 (95% CI, 0.4-49.6).

The ASQ identified 1229 patients (54.8% of positive ASQ screens) whose suicidal thoughts or behaviors would have been undetected through standard hospital procedures, particularly male patients and black patients. A previous study of universal screening in adult EDs yielded a similar rate and demographic distribution of undetected suicide risk.^17^ The improved detection of suicide risk in black youth is especially important given that the suicide rate has increased among elementary school–aged black youth over the past few decades.^3^ Conversely, 14.8% of those with an index presenting problem related to suicide risk screened negative on that same visit, which may represent the subset of youth who come to the ED for suicide-related reasons but either are no longer experiencing these thoughts by the time the ASQ is administered or chose not to speak about their suicidal ideation or behavior.

### Implementation Considerations

The direct comparison of selective vs universal screening and the analysis of psychiatric and nonpsychiatric subgroups within the universal subsample can inform best practices around suicide risk screening in pediatric EDs. Our finding that the HR was greatest for universal screening, particularly for universal screening of patients with nonpsychiatric presenting problems, takes The Joint Commission’s 2016 Sentinel Decree^10^ that health care professionals should use selective screening procedures 1 step further to show the value of universal screening. While universal screening is more labor intensive and can seem to be not clinically indicated or possibly irrelevant to medical patients, suicide risk screening was contextualized by nurses at triage as a patient safety effort and, overall, most clinicians in this ED were able to include the ASQ in their triage routines. Our results indicate that the ASQ was strongly associated with suicide risk among youth with medical or surgical presenting problems, which is particularly important as these patients may be more likely to slip through the cracks when detecting suicide risk and connecting to mental health care. Notably, our finding that the effect size for suicide-related presenting problems was very small after adjusting for ASQ results suggests that index presenting problems are a weak and insufficient indicator of subsequent suicidal behavior compared with suicide risk screening, at least when used in isolation.

### Strengths and Limitations

Strengths of this study include real-world comparison of the consecutive implementation of selective and universal screening approaches within the same setting as routine care. The availability of death records to supplement the hospital-based outcome measures was also a strength. However, one limitation was that the death records yielded an insufficient number of cases for reliable analyses and were limited to in-state deaths. We intend to revisit this outcome pending a longer follow-up period. We did not have access to data on mental health treatment after screening and thus could not study whether identification of suicide risk by screening resulted in greater engagement in mental health services and reduced future risk for suicidal behaviors.

An additional limitation is that medical records indicate that some medical and surgical patients were given the ASQ in the selective screening condition even though the protocol specified that it was to be given only to patients with presenting psychiatric problems. This may have inflated the HR (4.8) for the selective screening condition (based on comparison with the HR for the psychiatric-only subsample of the universal screening condition [2.2]), although this may more accurately reflect the real-world implementation of such screening tools, which may not be done with complete fidelity, particularly in busy ED settings. Furthermore, at least some of this variance from protocol was due to clinical judgment, as nursing staff sometimes administered the ASQ in the selective screening condition to medical and surgical patients who they felt to be at risk, although this was not consistently documented. Some youths were not screened even though they met inclusion criteria, which likewise reflects our use of a real-world ED setting for this study; youth sometimes cannot be fully assessed in ED triage due to aggressive behavior, cognitive issues, lack of responsiveness, or urgent need for medical attention, while other missed screens may have simply been due to oversight. Follow-up data were not available for those who were not screened, who may have varied from the screened samples in terms of subsequent risk for suicidal behavior.

This is also a single-site study, and we do not have data on patients who went to another hospital for subsequent ED visits. Because the selective and universal protocols were implemented consecutively as routine care based on a hospital policy change, the selective screening group had a longer follow-up period. We chose to include all participants rather than set a minimum follow-up period for the survival analyses, although a sensitivity analysis limiting the sample to individuals with at least 3 months of follow-up yielded similar results. Also, real-world implementation factors, such as the parents being present for some ASQ assessments, may have negatively affected the reliability of patient reports while simultaneously enhancing the external validity of the study.

## Conclusions

These data provide convincing evidence that implementing standardized suicide screening in a pediatric ED is an effective means of identifying otherwise undetected risk for subsequent suicidal behavior. Although screening with the ASQ was associated with future suicidal behaviors, enhanced follow-up, care coordination, or other community-based prevention approaches are needed to prevent future suicidal behaviors in our patient population.^19,20,21,22,23^ While the benefit in detection was greater for universal screening, both universal and selective screening resulted in improvements in detection of risk compared with treatment as usual. The increased detection of suicidal behavior among black youth may help address some emerging racial disparities in childhood suicide rates.^3^

## Notes

Supplement. **eAppendix 1.** ASQ Questions **eAppendix 2.** MD-SPIN Procedure **eAppendix 3.** Relative Risk for Death by Suicide **eFigure.** Demographic Comparisons Between Selective and Universal Sub-samples **eTable 1.** Cox Proportional Hazards Models, Testing ASQ Screens as Predictors of Subsequent Suicide-Related Outcomes Among Each Universal Sub-sample **eTable 2. **Frequency Data for Relative Risk Calculations Based on Positive ASQ Screens **eTable 3.** Frequency Data for Relative Risk Calculations, Based on Chief Complaint at Index Visit **eTable 4.** Frequency Data for Relative Risk Calculations, Using an “Either/Or” Approach in Which Suicide Risk Is Defined as a Positive ASQ Screen and/or a Suicide-Related Presenting Problem

## References

[1] Centers for Disease Control and Prevention. Ten leading causes of death by age group, United States—2017. https://www.cdc.gov/injury/wisqars/LeadingCauses.html. Published March 2019. Updated April 10, 2019. Accessed May 1, 2019.

[2] Hedegaard H, Curtin SC, Warner M Suicide Mortality in the United States, 1999–2017: NCHS Data Brief No. 330. Hyattsville, MD: National Center for Health Statistics; 2018.

[3] Bridge JA, Asti L, Horowitz LM, Suicide trends among elementary school–aged children in the United States from 1993 to 2012. JAMA Pediatr. 2015;169(7):-. doi:10.1001/jamapediatrics.2015.0465 25984947

[4] Caine ED Forging an agenda for suicide prevention in the United States. Am J Public Health. 2013;103(5):822-829. doi:10.2105/AJPH.2012.301078 23488515 PMC3698817

[5] Wahlbeck K Public mental health: the time is ripe for translation of evidence into practice. World Psychiatry. 2015;14(1):36-42. doi:10.1002/wps.20178 25655149 PMC4329888

[6] Horowitz LM, Ballard ED, Pao M Suicide screening in schools, primary care and emergency departments. Curr Opin Pediatr. 2009;21(5):620-627. doi:10.1097/MOP.0b013e3283307a89 19617829 PMC2879582

[7] Olfson M, Marcus SC, Bridge JA Focusing suicide prevention on periods of high risk. JAMA. 2014;311(11):1107-1108. doi:10.1001/jama.2014.501 24515285

[8] Appleby L, Shaw J, Amos T, Suicide within 12 months of contact with mental health services: national clinical survey. BMJ. 1999;318(7193):1235-1239. doi:10.1136/bmj.318.7193.1235 10231250 PMC27859

[9] Olfson M, Wall M, Wang S, Short-term suicide risk after psychiatric hospital discharge. JAMA Psychiatry. 2016;73(11):1119-1126. doi:10.1001/jamapsychiatry.2016.2035 27654151 PMC8259698

[10] The Joint Commission. Sentinel Event Alert: detecting and treating suicide ideation in all settings. https://www.jointcommission.org/assets/1/18/SEA_56_Suicide.pdf. Published February 24, 2016. Accessed May 1, 2019. 26915165

[11] Kumasaka Y, Stokes J, Gupta RK Criteria for involuntary hospitalization. Arch Gen Psychiatry. 1972;26(5):399-404. doi:10.1001/archpsyc.1972.01750230009002 5019875

[12] Large M, Kaneson M, Myles N, Myles H, Gunaratne P, Ryan C Meta-analysis of longitudinal cohort studies of suicide risk assessment among psychiatric patients: heterogeneity in results and lack of improvement over time. PLoS One. 2016;11(6):e0156322. doi:10.1371/journal.pone.0156322 27285387 PMC4902221

[13] Habis A, Tall L, Smith J, Guenther E Pediatric emergency medicine physicians’ current practices and beliefs regarding mental health screening. Pediatr Emerg Care. 2007;23(6):387-393. doi:10.1097/01.pec.0000278401.37697.79 17572523

[14] Horowitz LM, Bridge JA, Teach SJ, Ask Suicide-Screening Questions (ASQ): a brief instrument for the pediatric emergency department. Arch Pediatr Adolesc Med. 2012;166(12):1170-1176. doi:10.1001/archpediatrics.2012.1276 23027429 PMC6889955

[15] Ballard ED, Cwik M, Van Eck K, Identification of at-risk youth by suicide screening in a pediatric emergency department. Prev Sci. 2017;18(2):174-182. doi:10.1007/s11121-016-0717-5 27678381 PMC5247314

[16] Lanzillo EC, Horowitz LM, Wharff EA, Sheftall AH, Pao M, Bridge JA The importance of screening preteens for suicide risk in the emergency department. Hosp Pediatr. 2019;9(4):305-307. doi:10.1542/hpeds.2018-0154 30858170 PMC6434973

[17] Boudreaux ED, Camargo CA Jr, Arias SA, Improving suicide risk screening and detection in the emergency department. Am J Prev Med. 2016;50(4):445-453. doi:10.1016/j.amepre.2015.09.029 26654691 PMC4801719

[18] Spirito A, Lewander WJ, Levy S, Kurkjian J, Fritz G Emergency department assessment of adolescent suicide attempters: factors related to short-term follow-up outcome. Pediatr Emerg Care. 1994;10(1):6-12. doi:10.1097/00006565-199402000-00003 8177812

[19] Carter GL, Clover K, Whyte IM, Dawson AH, D’Este C Postcards from the EDge project: randomised controlled trial of an intervention using postcards to reduce repetition of hospital treated deliberate self poisoning. BMJ. 2005;331(7520):805. doi:10.1136/bmj.38579.455266.E0 16183654 PMC1246077

[20] Carter GL, Clover K, Whyte IM, Dawson AH, D’Este C Postcards from the EDge: 24-month outcomes of a randomised controlled trial for hospital-treated self-poisoning. Br J Psychiatry. 2007;191:548-553. doi:10.1192/bjp.bp.107.038406 18055960

[21] Fleischmann A, Bertolote JM, Wasserman D, Effectiveness of brief intervention and contact for suicide attempters: a randomized controlled trial in five countries. Bull World Health Organ. 2008;86(9):703-709. doi:10.2471/BLT.07.046995 18797646 PMC2649494

[22] Vaiva G, Vaiva G, Ducrocq F, Effect of telephone contact on further suicide attempts in patients discharged from an emergency department: randomised controlled study. BMJ. 2006;332(7552):1241-1245. doi:10.1136/bmj.332.7552.1241 16735333 PMC1471935

[23] Motto JA, Bostrom AG A randomized controlled trial of postcrisis suicide prevention. Psychiatr Serv. 2001;52(6):828-833. doi:10.1176/appi.ps.52.6.828 11376235

