# Supplementary material for: Assessment of Selective and Universal Screening for Suicide Risk in a Pediatric Emergency Department
Source: JAMA Netw Open. 2019 Oct 25;2(10):e1914070. doi: 10.1001/jamanetworkopen.2019.14070 (PMC6822088; doi:10.1001/jamanetworkopen.2019.14070)
Supplement: Supplement. — eAppendix 1. ASQ Questions eAppendix 2. MD-SPIN Procedure eAppendix 3. Relative Risk for Death by Suicide eFigure. Demographic Comparisons Between Selective and Universal Sub-samples eTable 1. Cox Proportional Hazards Models, Testing ASQ Screens as Predictors of Subsequent Suicide-Related Outcomes Among Each Universal Sub-sample eTable 2. Frequency Data for Relative Risk Calculations Based on Positive ASQ Screens eTable 3. Frequency Data for Relative Risk Calculations, Based on Chief Complaint at Index Visit eTable 4. Frequency Data for Relative Risk Calculations, Using an “Either/Or” Approach in Which Suicide Risk Is Defined as a Positive ASQ Screen and/or a Suicide-Related Presenting Problem [file jamanetwopen-2-e1914070-s001.pdf]

Supplementary Online Content

DeVylder JE, Ryan TC, Cwik M, et al. Assessment of selective and universal screening for suicide risk in a pediatric emergency department. *JAMA Netw Open*. 2019;2(10):e1914070. doi:10.1001/jamanetworkopen.2019.14070

eAppendix 1. ASQ Questions
eAppendix 2. MD-SPIN Procedure
eAppendix 3. Relative Risk for Death by Suicide
eFigure. Demographic Comparisons Between Selective and Universal Sub-samples
eTable 1. Cox Proportional Hazards Models, Testing ASQ Screens as Predictors of Subsequent Suicide-Related Outcomes Among Each Universal Sub-sample
eTable 2. Frequency Data for Relative Risk Calculations Based on Positive ASQ Screens
eTable 3. Frequency Data for Relative Risk Calculations, Based on Chief Complaint at Index Visit
eTable 4. Frequency Data for Relative Risk Calculations, Using an "Either/Or" Approach in Which Suicide Risk Is Defined as a Positive ASQ Screen and/or a Suicide-Related Presenting Problem

This supplementary material has been provided by the authors to give readers additional information about their work.

© 2019 DeVylder JE et al. *JAMA Network Open.*

eAppendix 1: ASQ Questions

Patients are asked the following four questions: “In the past few weeks, have you wished you were dead?”, “In the past few weeks, have you felt that you or your family would be better off if you were dead?”, “In the past week, have you been having thoughts about killing yourself?”, and “Have you ever tried to kill yourself?”

© 2019 DeVylder JE et al. *JAMA Network Open.*

eAppendix 2. MD-SPIN PROCEDURE

As a way to comply with the Joint Commission's recommendation and in an attempt to address the growing rate of youth suicide, a pediatric emergency department in an urban hospital implemented suicide risk screening as a standard of care.

The implementation of systematic suicide risk screening began with the formation of a task force consisting of nurses, doctors, researchers, and other stakeholders. The task force's first objective was to select a suicide risk-screening tool; the committee wanted to select a tool that had strong psychometrics and that was both brief and easy to implement. Based on the latter criteria the group decided to use to Ask Suicide-Screening Questions tool better known as the ASQ[13]. Early studies on the ASQ indicated that the screener had good psychometrics with high sensitivity and specificity.

After the screening tool was selected, medical staff were given training on how to administer the ASQ and talk to patients about suicide. The ASQ screening tool was then built into the electronic health record, making it simple for nursing staff to administer and record in the busy ED setting. Due to concerns surrounding workflow and acuity, the ASQ was initially just administered to youth ages 8 to 18 presenting to the pediatric ED with a psychiatric chief complaint.

The medical staff was able to implement the ASQ with relatively little interruption of the ED workflow, and found that the ASQ was valuable in recognizing patients who may be at an increased risk for suicide. Due to the successful implementation of the ASQ in psychiatric patients, in January of 2017, the ED staff decided to implement universal suicide risk screening; this meant that every patient age eight and older would be screened with the ASQ. The nurses administered the ASQ along with the other routine screenings they conduct at triage. Screening at triage was ideal because than medical providers can prepare to adjust their plan of care if a positive screen occurs. If a patient did screen positive on the ASQ, the medical team was notified and the ED social work team conducted a risk assessment.

Data on ASQ screens were extracted monthly from the EHR. Data included the patient's age, race, ethnicity, sex, chief complaint, principle diagnosis, date of ED visit, health co-morbidities, ED disposition, and response to each of the ASQ items. When universal screening began in January of 2017, the same data was extracted from the universal population. A database was created for all ASQ screenings completed over the three and a half years. No patients were excluded on the basis of gender, race, or sex.

© 2019 DeVylder JE et al. *JAMA Network Open.*

Relative Risk for Death by Suicide

Relative risk for death by suicide could be calculated only for the selective screening sample, as there were no deaths due to suicide recorded in the universal screening sample within our data. Similarly, analysis of relative risk for death within the selective screening sample should be considered exploratory given the low total number of deaths by suicide. Relative risk is nonetheless presented in order to provide a preliminary estimate of the magnitude of association between positive screen and subsequent death by suicide within the selective screening sub-sample.

© 2019 DeVylder JE et al. *JAMA Network Open.*

eAppendix 3. Relative Risk for Death by Suicide

There were three deaths by suicide within the selective screening sub-sample during the follow-up period, as well as one additional undetermined death. Of these deaths, two of those that died by suicide had screened positive on the ASQ, and the third had screened negative. The undetermined death likewise screened negative. The relative risk for confirmed death by suicide in the selective screening subsample was RR(95% CI)=4.50(0.41-49.57), and with inclusion of the undetermined death, RR(95% CI)=2.25(0.32-15.96). There were no deaths by suicide among the universal screening sub-sample within our follow-up period. All three deaths occurred at least a year after the initial screening, M(SD)=833.33(210.07) days from the index visit.

Notably, of the three confirmed deaths by suicide, two had first come to the ED with chief complaints unrelated to suicide (i.e., wheezing and externalizing behaviors). While the youth with externalizing behaviors screened negative on the ASQ, the one with a chief complaint of wheezing screened positive, and had a documented history of being seen for homicidal ideation. Both of the patients who screened positive on the ASQ were admitted or transferred from the ED during this index visit, whereas the one who screened negative was discharged. Taken together, of these three deaths by suicide, suicide risk was identified at the index visit for one based on chief complaint and the ASQ, one by the ASQ alone (i.e., would have otherwise not been identified), and one was missed entirely, or perhaps did not yet experience suicidal ideations or behavior.

© 2019 DeVylder JE et al. *JAMA Network Open.*

eFigure. Demographic comparisons between selective and universal sub-samples. Selective and Universal sub-samples varied in terms of race, X2(4,n=15003)=118.89,p<0.001, gender, X2(1,n=15003)=12.92, p<0.001, and age, t(15001)=13.09, p<0.001.

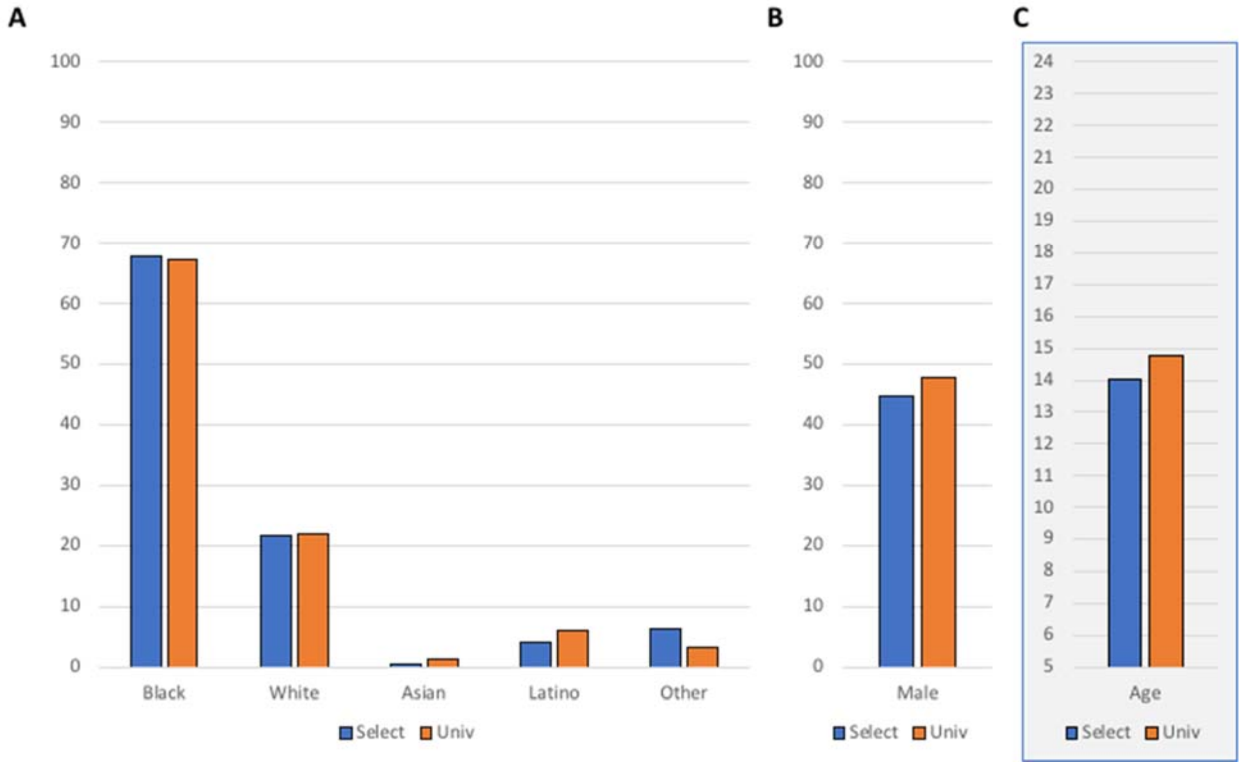

© 2019 DeVylder JE et al. *JAMA Network Open.*

eTable 1. Cox Proportional Hazards Models, testing ASQ screens as predictors of subsequent suicide-related outcomes among each universal sub-sample.

Variable	HR	95% CI	HR	95% CI
Universal Sample: psychiatric only				
ASQ				
Positive	**3.05**	**1.78-5.22**	**2.21**	**1.19-4.08**
Negative	1.00	-	1.00	-
Race				
Black, non-Latino	1.00	-	1.00	-
White, non-Latino	0.82	0.45-1.48	0.78	0.43-1.42
Asian, non-Latino	0.00	*na*	0.00	*na*
Latino	1.16	0.46-2.91	1.19	0.47-2.98
Other	0.00	*na*	0.00	*na*
Gender				
Male	1.01	0.62-1.64	1.03	0.63-1.68
Female	1.00	-	1.00	-
BLOCK 2				
Presenting problem				
Suicide-related			1.57	0.92-2.70
Other			1.00	-
Admittance				
Discharged			1.00	-
Admitted			1.51	0.92-2.49
Transferred/AMA/Other			2.04	0.28-15.00
Universal sample: non-psychiatric only				
ASQ				
Positive	**6.95**	**3.12-15.51**	**7.06**	**3.16-15.79**
Negative	1.00	-	1.00	-
Race				
Black, non-Latino	1.00	-	1.00	-
White, non-Latino	1.00	0.49-2.03	0.99	0.49-2.01
Asian, non-Latino	0.00	*na*	0.00	*na*
Latino	1.81	0.71-4.65	1.80	0.70-4.62
Other	1.32	0.32-5.53	1.31	0.31-5.48
Gender				
Male	0.60	0.33-1.08	0.59	0.33-1.06
Female	1.00	-	1.00	-
BLOCK 2				
Presenting problem				
Suicide-related			-	-
Other			-	-
Admittance				
Discharged			1.00	-
Admitted			1.22	0.59-2.53
Transferred/AMA/Other			0.00	*na*

© 2019 DeVylder JE et al. *JAMA Network Open.*

eTable 2. Frequency data for relative risk calculations based on positive ASQ screens.

Screening Results	Follow-up visit for suicidal behavior	No follow-up visit for suicidal behavior	Total
Selective screening			
ASQ+	77	1358	1435 (5.37)
ASQ-	14	3217	3231 (0.43)
Total	91	4575	4666
		RR (95% CI) = 12.38 (7.03-21.81)	
Universal screening			
ASQ+	27	664	691 (3.91)
ASQ-	18	8371	8389 (0.21)
Total	45	9035	9080
		RR (95% CI) = 18.21 (10.08-32.90)	
Combined (full sample)			
ASQ+	104	2022	2126 (4.89)
ASQ-	32	11588	11620 (0.28)
Total	136	13610	13746
		RR (95% CI) = 17.76 (11.98-26.33)	

Note: RR=Relative Risk; CI=Confidence Interval

© 2019 DeVylder JE et al. *JAMA Network Open.*

eTable 3. Frequency data for relative risk calculations, based on chief complaint at index visit.

Initial Presenting Problem	Follow-up visit for suicidal behavior	No follow-up visit for suicidal behavior	Total
Selective screening			
Suicidal Behavior	47	675	722 (6.51)
Other	44	3900	3944 (1.12)
Total	91	4575	4666
		RR (95% CI) = 5.84 (3.90-8.73)	
Universal screening			
Suicidal Behavior	19	362	381 (4.99)
Other	26	8673	8699 (0.30)
Total	45	9035	9080
		RR (95% CI) = 17.13 (9.57-30.68)	
Combined (full sample)			
Suicidal Behavior	66	1037	1103 (5.98)
Other	70	12573	12643 (0.55)
Total	136	13610	13746
		RR (95% CI) = 10.81 (7.77-15.04)	

Note: RR=Relative Risk; CI=Confidence Interval.

© 2019 DeVylder JE et al. *JAMA Network Open.*

eTable 4. Frequency data for relative risk calculations, using an "either/or" approach in which suicide risk is defined as a positive ASQ screen and/or a suicide-related presenting problem.

Presenting Problem or ASQ	Follow-up visit for suicidal behavior	No follow-up visit for suicidal behavior	Total
Selective screening			
Positive	80	1426	1506 (5.31)
Negative	11	3149	3160 (0.35)
Total	91	4575	4666
		RR (95% CI) = 15.26 (8.15-28.58)	
Universal screening			
Positive	29	734	763 (3.80)
Negative	16	8301	8317 (0.19)
Total	45	9035	9080
		RR (95% CI) = 19.76 (10.78-36.21)	
Combined (full sample)			
Positive	109	2160	2269 (4.80)
Negative	27	11450	11477 (0.24)
Total	136	13610	13746
		RR (95% CI) = 20.42 (13.43-31.05)	

Note: RR=Relative Risk; CI=Confidence Interval

© 2019 DeVylder JE et al. *JAMA Network Open.*
